# Exploring phytochemicals and their pharmacological applications from ethnomedicinal plants: A focus on *Lycium barbarum, Solanacea*

**DOI:** 10.1016/j.heliyon.2025.e41782

**Published:** 2025-01-09

**Authors:** Baiken Baimakhanova, Amankeldi Sadanov, Andrey Bogoyavlenskiy, Vladimir Berezin, Lyudmila Trenozhnikova, Gul Baimakhanova, Aibat Ibraimov, Elmira Serikbayeva, Zhalgaskali Arystanov, Tanagul Arystanova, Rakhym Nazakat, Ainura Khammetova, Gulnaz Seitimova, Aknur Turgumbayeva

**Affiliations:** a Research and Production Center for Microbiology and Virology LLC, Almaty, 050010, Kazakhstan; b School of Pharmacy, JSC “S.D. Asfendiyarov Kazakh National Medical University”, Almaty, 050000, Kazakhstan; c NJSC “Astana Medical University”, Astana, 010000, Kazakhstan; d Chemistry and Chemical Technology Faculty, Al-Farabi Kazakh National University, Almaty, 050040, Kazakhstan; e Higher School of Medicine, Al-Farabi Kazakh National University, Almaty, 050040, Kazakhstan

**Keywords:** *Lycium barbarum*, Anti-inflammatory, Neuroprotective, Antidiabetic, Polysaccharides

## Abstract

*Lycium barbarum* is a species commonly utilized in dietary supplements and natural healthcare products. *Lycium barbarum* also referred to as wolfberry or goji berry, are predominantly found in China, Japan, Korea, and North America. *Lycium barbarum* has a lengthy history as a medicinal and functional food. Recent studies showcasing their exceptional bioac-tive properties have led to an increase in their cultivation and popularity worldwide. *Lycium barbarum* have been recognized as a valuable source of functional ingredients with promising applications in the food and medical sectors. *Lycium barbarum* are rich in phytochemical compounds such as polysaccharides, carotenoids, organic acids, carbohydrates (fructose and glucose), phenolic compounds (such as phenolic acids and flavonoids), and vitamins (ascorbic acid). Various biological activities, including antimicrobial, anti-inflammatory, prebiotic, neuroprotective, antidiabetic, and gastroprotective effects, have linked to their consumption. The purpose of this review is to provide a comprehensive overview of the phytochemical components as well as the biological active properties of *Lycium barbarum.*

## 1 Introduction

**Fig. 1:**
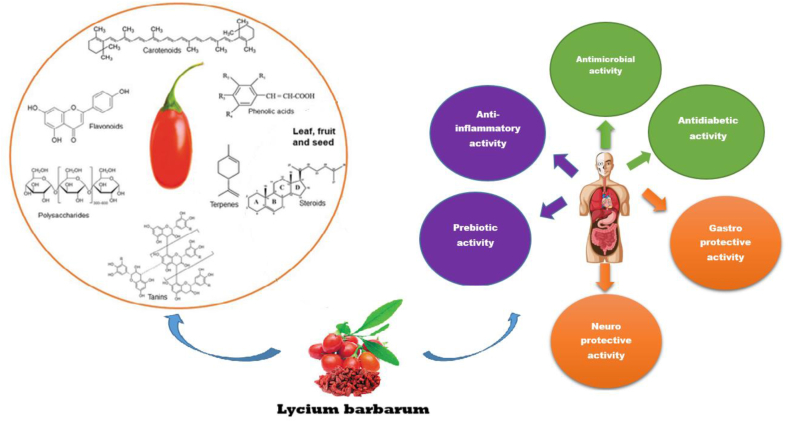
Pharmacological properties of *Lycium barbarum*.

Plants have been utilized for an extended period in traditional medicine to aid in the prevention and treatment of diseases [1]. One such plant is *Lycium barbarum*, also known as wolfberry and goji berry, which offers various health benefits. This plant belongs to the *Solanacea* family and is cultivated year-round in regions such as China, Japan, Korea, North America, Europe, and Central Asia [2] (see Fig. 1).

Out of the 97 *Lycium* genus species, 31 are used for both food and medicine [3]. *Lycium barbarum* can be classified into different groups based on their ripening stage, size, weight, color, firmness, sugar content, acidity level, and titratability [4]. Fully ripened fruits measure 1–2 cm in length, have an elliptical shape, and display a bright orange-red color, with each fruit containing up to 40 small seeds [[4], [5], [6]]. As the demand for *Lycium barbarum* fruits continues to rise, they have become a global market commodity. Currently, China leads the global market for *Lycium barbarum* fruits, with the majority of these berries being grown in the northwestern regions of China, including Ningxia, Xinjiang, Tibet, Inner Mongolia, Qinghai, and Gansu [7,8].The berries have a sweet taste and are widely used as a dietary supplement and natural remedy [3,10,11]. While they are primarily consumed fresh in their in places of growth, they are mostly consumed in their dried form globally, or processed into various food products such as juices, teas, yogurt, muesli, powders, and capsules [8,[12], [13], [14]].

Throughout the ages, *Lycium barbarum* has been utilized as a traditional remedy in Asian countries [15]. Renowned for its rich nutritional value and health benefits, such as antioxidant, antimicrobial, immune-boosting, and anti-inflammatory properties [9,10,16], the fruit is valued for its anti-aging effects, soothing properties, and thirst-quenching abilities [6,17]. In ancient healing traditions, the berries of *Lycium barbarum* were employed by communities to enhance blood circulation, address early diabetes, tuberculosis, dizziness, and persistent cough, as well as protect eye health [18]. In modern medical studies, the wide-ranging advantages of *Lycium barbarum* have been confirmed, including anti-cancer, anti-inflammatory effects, and promotion of gut health at the cellular or animal level [2,7,12].

Various natural factors, including weather conditions, rainfall, soil texture, and salt content, contribute to the development of diverse bioactive compounds [[19], [20], [21]]. Various bioactive components, such as polysaccharides, carotenoids, vitamins, flavonoids, alkaloids, amino acids and fatty acids, have been isolated and identified from the fruits of *Lycium barbarum*. Among these, polysaccharides are particularly noteworthy, constituting 5–8% of the dried fruits [2]. Over the past few decades, numerous studies have supported the idea that *Lycium barbarum* polysaccharides (LBPs) possess a variety of biological properties, including immunoregulation, anti-inflammatory effects, antitumor properties, hypoglycemic/lipidemic effects, and protection of the retina [[22], [23], [24]]. Therefore, the aim of this work is to provide a comprehensive overview of the bioactive compounds of *Lycium barbarum* as well as their biological active properties, which will serve in the future as an important source in the study of the plant of *Lycium barbarum.*

## 2 Chemical components

### 2.1 Polysaccharides

*Lycium barbarum* is rich in polysaccharides (LBP) as its main active ingredient. The sugar content of *Lycium barbarum* accounts for approximately 40 % of its dry weight, with LBP making up 5–8 % of the total dry weight. Various extraction techniques, including cold or hot water extraction, microwave extraction, enzymatic extraction, ultrasonic extraction, and supercritical fluid extraction, are employed to extract LBP [2,25]. Cold water extraction is the preferred method for obtaining crude LBP due to its simplicity and high efficiency [13,25]. High molecular weight polysaccharides have been successfully extracted from *Lycium barbarum* through cold water extraction, with yields ranging from 2 to 3 % [13]. Further improvements in yields can be achieved through prolonged high-temperature extraction or enzymatic processing. Some studies suggest that an increase in LBP content results in higher concentrations of pectin, cellulose, and hemicellulose polysaccharides during extended processing methods such as high temperature, enzymatic treatment, and microwave treatment [16,26]. Water-soluble extracts obtained through the aforementioned extraction methods often contain impurities like inorganic salts, pigments, monosaccharides, oligosaccharides, and proteins, which can impact the analysis of the LBP structure. To address this issue, purification methods such as membrane separation, column chromatography, and chemical precipitation are utilized [25,27]. Moreover, the phenol-sulfuric acid method indicated that hot water extraction and microwave-ultrasonic synergistic extraction methods resulted in polysaccharide extraction rates of 6.71 % and 9.62 %, respectively [28]. By optimizing with response surface method and orthogonal test, the highest polysaccharide recovery reached 13.25 % using the amylase method for LBP extraction and phenol-sulfuric acid method for content determination [29]. When ultrasound and microwave irradiation were combined for extraction, the polysaccharide yield was 1.873 % under optimized conditions [30].

Currently, over 30 polysaccharides have been extracted and characterized from Lycium barbarum. Analysis of the monomeric composition of these polysaccharides revealed that they primarily consist of nine different monosaccharides: mannose (Man), rhamnose (Rha), galactose (Gal), xylose (Xyl), arabinose (Ara), fucose (Fuc), glucose (Glc), galacturonic acid (GalA), and glucuronic acid (GlcA). The structural backbones of the polysaccharides from Lycium barbarum are predominantly made up of (1 → 4)-β-Galp, (1 → 3)-β-Galp, (1 → 6)-α-glucans, (1 → 6)-β-Galp, and (1 → 4)-α polygalacturonans, featuring various branches and terminal structures. Additionally, the glycan derived from L. barbarum has a backbone primarily consisting of (1 → 6)-β-galactosyl residues, with approximately 50 % of these residues substituted at C-3 by either galactosyl or arabinosyl groups. Furthermore, the backbone of an acidic polysaccharide isolated from L. barbarum, is mainly composed of (1,4)-linked galacturonic acid, (1,5)-linked arabinose, and (1)-mannose-(3,6)-linked structures, concluding with a terminal (1)-mannose. Additionally, the backbone of arabinogalactan-proteins (AGPs) identified from L. barbarum consists of (1 → 3)-linked β-D-galactopyranosyl residues, with predominantly 5-substituted α-L-arabinofuranosyl residues at O-6 as side chains. Notably, AGPs exhibit a unique structure featuring 4-substituted β-D-glucopyranosyluronic acid residues within their side chains [2,13,16], [[25], [26], [27], [28], [29], [30]].

### 2.2 Polyphenols

Various techniques used to detect polyphenols in *Lycium barbarum* include high-performance liquid chromatography (HPLC), gas chromatography-mass spectrometry (GC-MS), and ultra-performance liquid chromatography-triple quadrupole mass spectrometry (UPLC-TQ-MS). Flavonoids such as rutin, quercetin, kaempferol, myricetin, hesperidin, and naringin have been identified. Phenolic acids like chlorogenic acid, p-coumaric acid, ferulic acid, protocatechuic acid, and gallic acid are also present. The characterization of eleven phenolic compounds from *Lycium barbarum* using liquid chromatography-high resolution mass spectrometry (LC-HRMS/QTOF) includes chlorogenic acid, esculetin, caffeic acid, rutin, ellagic acid, p-hydroxycinnamic acid, scopoletin, 5,7-dihydroxy-4-methylcoumarin, morin, quercetin, and curcumin [31]. A recent research revealed that five significant phenolic compounds were found and successfully identified through HPLC-MS analysis, such as rutin, p-coumaric acid, and its hexoside derivatives [32].

### 2.3 Carotenoids

Recently, scientists have identified carotenoids in *Lycium barbarum*, finding zeaxanthin, β-carotene, and zeaxanthin dipalmitate. Zeaxanthin dipalmitate had levels between 0.81 mg/g and 4.05 mg/g, while zeaxanthin ranged from 5.88 to 28.17 μg/g, and β-carotene was close to the threshold value [33]. Carotenoids were also detected in *Lycium barbarum* from five locations, revealing zeaxanthin dipalmitate, zeaxanthin, and β-carotene. Zeaxanthin dipalmitate had the highest concentration at 21.03 mg/g, followed by zeaxanthin, with β-carotene having the lowest content at 0.01 mg/g [34]. Aneta et al. used LC-QTOF-MS to identify zeaxanthin, β-carotene, neoxanthin, and cryptoflavin in *Lycium barbarum*. Zeaxanthin had the highest content at an average of 845.39 mg/kg, while neoxanthin had the lowest content at an average of 160.35 mg/kg [35]. Xu et al. found nine components in *Lycium barbarum* carotenoid extract using HPLC, including all-trans-zeaxanthin and its isomers, all-trans-β-carotene and its isomers, neoxanthin, and β-cryptoxanthin. The maximum content of all-trans-zeaxanthin and its isomers was 1666.3 μg/g, and the minimum content of neoxanthin was 4.47 μg/g [[36], [37]]. Carotenoids in *Lycium barbarum* can be in free or ester form, influencing the color of the fruit. An analysis showed that red *Lycium barbarum* have a higher total carotenoid content compared to yellow *Lycium barbarum*, ranging from 39.74 to 54.76 mg/100 g [38]. The level of carotenoids was in direct correlation with the redness of the *Lycium barbarum*. A separate analysis using colorimetry revealed that the red *Lycium barbarum* had the highest carotenoid content at 233.08 μg/g, while the *black Lycium* had the lowest at 1.51 μg/g. As a result, the carotenoid content ranked in the order of red *Lycium barbarum*, yellow *Lycium barbarum*, and *black Lycium* [15].

### 2.4 Vitamins

*Lycium barbarum* is rich in vital vitamins including vitamin A (VA), vitamin B1 (VB1), vitamin B2 (VB2), and vitamin C (VC). The VC content in 21 different *Lycium barbarum* varieties ranged from 2.39 to 6.24 mg/100 g as analyzed by UPLC-PDA. Among them, g43 had the highest VC levels while g22 had the lowest [35]. In a different research, VC levels were assessed in two *Lycium barbarum* species (Lasa and New Big) cultivated in Lithuania using the titration method. The highest vitamin C concentration was detected in New Big at 5.8 mg/100 g FW [39]. In terms of VB1 content, cultivated *Lycium barbarum* had an average of 7.87 μg/g, while wild *Lycium barbarum* had 7.10 μg/g, as determined by the fluorescent method. The cultivated *Lycium barbarum* exhibited higher VB1 content, possibly due to superior growth conditions and sufficient water supply [40]. Examination of VB1 and VB2 content in *black Lycium* and red hijo berries utilizing the UPLC method showed that *black Lycium* contained 21.7 mg/kg of VB1 and 17.6 mg/kg of VB2, while red *Lycium barbarum* had 9.4 mg/kg of VB1 and 4.5 mg/kg of VB2. The VB2 content in *black Lycium* surpassed that in red *Lycium barbarum* [41]. The levels of VA and VE in *black Lycium* oil were determined using HPLC, with VA at 0.3 mg/100 g and VE at 46.3 mg/100 g [42].

### 2.5 Others

Moreover, aside from the active ingredients mentioned earlier, *Lycium barbarum* is rich in amino acids, vitamins, trace elements, and other essential components crucial for maintaining optimal health (Table 1). An amino acid analysis revealed that *Lycium barbarum* berries contain 16 different amino acids, with aspartic acid being the most abundant at 3.00 mg/g [43]. When comparing the amino acid content of red, black, and yellow *Lycium barbarum*, it was found that *black Lycium* had the highest amino acid content at 9.04 g/100 g. *Black Lycium* also boast significant levels of magnesium, calcium, iron, manganese, copper, and zinc, with iron having the highest concentration at 36.1 mg/100 g [44]. Furthermore, using size exclusion chromatography coupled with inductively coupled plasma mass spectrometry, trace elements like manganese, iron, copper, zinc, selenium, and molybdenum were detected in *Lycium barbarum*. Zinc was found to have the highest content at 10.6 μg/g, while manganese and copper had contents of 9.9 μg/g and 6.1 μg/g, respectively[45].

**Table 1:** Major biologically active compounds of *Lycium barbarum*.

Extracts	Plant Part	Quality of control	Bio-active compounds	Pharmacology activity	Country	Reference
			Polysaccharides			
Water, ethanol	Fruit	FT-IR	Arabinose, galactose, rhamnose, glucose, galacturonic acid, mannose, glucosamine, xylose, fructose, ribose	Immunomodulatory and Anti-inflammatory	China	[11]
Ethanol	Fruit	HPLC	Arabinose, galactose, glucuronic acid, rhamnose,	Antitumor and Angiogenesis	China	[46]
Etanolic, Metanolic	Fruit	HPLC	Galactose, arabinose	Neuroprotective	China	[15]
Etanolic, acetone	Fruit	HPLC	Arabinose, galactose, rhamnose, gluconic acid, glucose, galacturonic acid, fructose, xylose	Hypoglycemic	China	[13]
Metanolic	Berry	HPLC	Arabinose, galactoaluronic acid, glucose	Hypoglycemic	China	[47]
Etanolic, ethyl acetate	Berry	UHPLC–MS/MS	Arabose, galactose, rhamnose, galactosamine, gluconic acid, glucose, xylose, ribose, mannose	Immunomodulatory	China	[16]
Etanolic, n-hexane	Fruit	LC–QTOF-MS	Arabinose, glucose, galactose	Antioxidant, Anti-inflammatory and Immunomodulatory	China	[27]
Ethyl acetate	Leaves	LC–ESI-MS/MS	Arabose, galactose, rhamnose, xylose, glucose, mannose	Renal protection	China	[26]
Etanolic, n-hexane	Leaves	UPLC–MS	Arabinose, galactose, rhamnose, xylose, galacturonic acid	Antioxidant and Immunomodulatory	China	[16]
			Polyphenols			
Deep eutectic solvents	Fruits	HPLC	Myricetin (57.2 mg/g), rutin (9.1 mg/g), mul-berin (12.7 mg/g),	Antioxidant, antibacterial and Anti-amyloidogenic	China	[48]
Deep eutectic solvents	Berry	HPLC	Quercetin (369.8 ± 44.5 μg/g), myricetin (117.3 ± 4.9 μg/g), kaempferol (93.6 ± 6.7 μg/g), quercetin-rhamnose-dihexoside (1065 ± 65.3 μg/g), rutin (76.1 ± 8.3 μg/g), quercetin-3-*O*-rubutin (628.9 ± 21.5 μg/g)	Antioxidant, antimicrobial, Cardiovascular and Antitumor	China	[49]
Deep eutectic solvents	Fruits	UHPLC–MS/MS	Rutin (1947 μg/g), aurantiamarin (9.50 μg/g), naringin (2.03 μg/g), neohesperidin (8.49 μg/g), hesperetin (1.32 μg/g), naringenin (52.2 μg/g)	Anti-allergic, Cardiovascular and anti-inflammatory	China	[50]
Trolox	Fruits	UPLC–TQ-MS	Kaempferol-3-*O*-rubutin (1066.02 ± 0.44 μg/g), rutin (7229.32 ± 0.12 μg/g), neochlorogenic acid (3.13 ± 0.02–574.21 ± 0.25 μg/g), p-hydroxybenzoic acid (6.79 ± 3.51–190.08 ± 0.85 μg/g), protocatechualdehyde (0.87 ± 0.00–9.47 ± 0.06 μg/g), p-coumaric acid (1.64 ± 3.77–67.70 ± 0.27 μg/g), caffeic acid (0.58 ± 0.05–19.37 ± 2.55 μg/g), chlorogenic acid (77.07 ± 12.33–10,203.92 ± 1.96 μg/g), cryptochlorogenic acid (12.33 ± 2.80–961.93 ± 11.23 μg/g), ferulic acid (3.10 ± 0.48–19.55 ± 0.32 μg/g)	Antimicrobial and Neuroprotective	China	[51]
Ethanol	Berry	UPLC–Q-Orbitrap MS	Rutin (143.98 ± 62.1 μg/g), quercetin (4.02 ± 0.12 μg/g), myricetin (4.56 ± 0.15 μg/g), naringenin (0.98 ± 0.02 μg/g), kaempferol (0.78 ± 0.05 μg/g), gallate (13.5 ± 0.17 μg/g), chlorogenic acid (162.66 ± 24.34 μg/g), catechin (5.46 ± 0.13 μg/g), vanillic acid (2.88 ± 0.08 μg/g), syringic acid (1.02 ± 0.01 μg/g), caffeic acid (119.7 ± 21.65 μg/g), p-coumaric acid (554.4 ± 38.7 μg/g), salicylic acid (2.41 ± 0.07 μg/g), ferulic acid (114.54 ± 15.7 μg/g), gallogen (4.5 ± 0.14 μg/g)	Antioxidant, Cardiovascular and Neuroprotective	Tibetan plateau, China	[52]
Ultrasound-assisted extraction	Fruits	HPLC	Chlorogenic acid (6.48 ± 0.16 mg/g), syringic acid (0.15 ± 0.01 mg/g), caffeic acid (1.41 ± 0.043 mg/g), ferulic acid (1.17 ± 0.04 mg/g), p-coumaric acid (0.83 ± 0.03 mg/g),	Anti-diabetic, Anti-melanogenic, Anti-tumor and Gastroprotective	Dulan, China	[53]
Ethyl acetate	Fruits	UPLC–MS	Salicylic acid (1.8 ± 0.1–2.3 ± 0.4 ng/mg), syringic acid (0.3 ± 0.1–0.9 ± 0.1 ng/mg), 4-hydroxybenzoic acid (7.8 ± 0.1–8.1 ± 0.3 ng/mg), p-coumaric acid (6.8 ± 0.1–178.4 ± 9.3 ng/mg), gallate (1.2 ± 0.1–1.9 ± 0.3 ng/mg), vanillic acid (1.8 ± 0.1–26.4 ± 0.3 ng/mg), ferulic acid (31.3 ± 0.3–33.6 ± 3.6 ng/mg), caffeic acid (0.7 ± 0.1–2.5 ± 0.3 ng/mg), protocatechuic acid (0.7 ± 0.1–1.0 ± 0.1 ng/mg)	Antimicrobial, Anti-inflammatory and Antidiabetic	China	[54]
Ethanol	Fruits	LC–ESI-MS/MS	Protocatechuic acid (91.6 ± 0.4 ng/g), gentisic acid (18.2 ± 0.0 ng/g), trans caffeic acid (46.4 ± 0.1 ng/g), p-coumaric acid (1644.1 ± 3.5 ng/g), isoferulic acid (9120.1 ± 3.1 ng/g), ferulic acid (684.2 ± 2.4 ng/g), hydroxybenzoic acid (664.3 ± 3.2 ng/g), salicylic acid (508.4 ± 2.2 ng/g)	Anti-allergenic, Immunoregulatory and Antithrombotic	Poland	[55]
			Carotenoids			
Methanol, dichloromethane	Fruits	HPLC	Zeaxanthin (28.17 μg/g), zeaxanthin dipalmitate (0.81–4.05 mg/g), β-carotene (5.62–8.04 μg/g),	Antiparasitic, and Antiosteoporosis effects	China	[56]
Ethanol	Berry	HPLC	Zeaxanthin dipalmitate (21.03 mg/mL), β-carotene (0.01 mg/mL), zeaxanthin (0.14 mg/mL)	Antioxidant and Anthelmintic	China	[57]
Ethanol, methanol	Berry	LC–QTOF-MS	Zeaxanthin (845.39 mg/kg), neoxanthin (160.35 mg/kg), β-carotene (193.53 mg/kg), cryptoflavin (722.94 mg/kg)	Antioxidant and Antiosteoporosis	Poland	[58]
Hexane–ethanol–acetone	Fruits	HPLC	All-trans zeaxanthin and its three isomers (1666.3 μg/g), neoxanthin (4.47 μg/g), β-cryptoxanthin (51.69 μg/g), all-trans β-carotene and its two isomers (20.11 μg/g)	Antioxidant, antiobesity and anti-inflammatory	China	[59]
Ethanol	Fruits	HPLC	(3R, 3′S)-zeaxanthin (0.522 μg/mL), (3R, 3′R, 6′R)-lutein (0.582 μg/mL), (3R, 3′R)-zeaxanthin (0.398 μg/mL),	Antiparasitic, anti-arthrisits and anti-inflammatory	China	[60]
Ethanol, methanol	Fruits	HPLC–PDA-MS	Zeaxanthin (0.6 ± 0.2 %), zeaxanthin palmitate (3.4 ± 0.2 %), β-carotene (0.8 ± 0.2 %), β-cryptoxanthin palmitate (5.1 ± 1.1 %), zeaxanthin myristate palmitate (1.9 ± 0.4 %), antheraxanthin dipalmitate (1.0 ± 0.2 %), zeaxanthin palmitate stearate (1.1 ± 0.1 %), zeaxanthin dipalmitatec (80.4 ± 0.6 %),	Antioxidant and Antiparasitic	Germany	[61]
Ethanol, ethyl acetate	Fruits	UPLC–MS	β-carotene (0.02–7.9 μg/g), lycopene (0.1–22.0 μg/g), lutein (0.2–97.5 μg/g), zeaxanthin (0.02–14.2 μg/g), violaxanthin (0.1–47.7 μg/g), zeaxanthin dipalmitate (0.2–94.2 μg/g)	Antioxidant and anti-inflammatory	France	[62]
Ethanol, water, acetone	Berry	HPLC–DAD	Zeaxanthin dipalmitate (4.5–5.5 mg/g)	Antioxidant and anti-inflammatory	Italy	[63]
Methanol, acetone	Fruits	HPLC	Zeaxanthin dipalmitate (211.4 mg/100 g), β-carotene (1.2 mg/100 g), zeaxanthin dipalmitate esters (37.5 mg/100 g)	Antioxidant, Antiparasitic and anti-inflammatory		[64]
			Vitamins			
Methanolic, water	Fruits	HPLC	Tocopherol (0.33 mg/100 g dw), ascorbic acid (2.39–48.94 mg/100 g fw),	Antioxidant	Italy	[6, 13]
			Fatty acids			
Ethanol, methanol	Fruits	HPLC	Linoleic acid (37.89–53.4 %), palmitic acid (12.77–21.79 %), oleic acid (16.5–23.6 %),	Anti-obesity, neuroprotection and anti-metabolic	Spain	[13,65],

## 3 Antimicrobial activity

The research on *Lycium barbarum* extracts from Eastern Europe revealed promising results in fighting against both gram-positive and gram-negative bacteria. The ethanol extract of *Lycium barbarum* exhibited strong antibacterial activity against *Bacillus subtilis* (1.8 ± 0.1) and *Staphylococcus aureus* (3.5 ± 0.1). Furthermore, *L. monocytogenes* and *S. typhimurium* strains were also susceptible to *Lycium barbarum* extracts, showing inhibition zones of 19–21 mm in diameter. The MIC values ranged from 50 to >100 μg/mL, with *S. typhimurium* being the most sensitive strain at 50 μg/mL, while *L. monocytogenes* was the least sensitive with an MIC value of >100 μg/mL. Additionally, Iraqi *Lycium barbarum* fruits exhibited similar antibacterial properties. The ethanolic extract of *Lycium barbarum* demonstrated effectiveness against both gram-positive and gram-negative bacterial strains, with an MIC value of 150 μg/mL [[66], [67], [68]].

The study conducted Skenderidis et al. found that ultrasonic water and ethanol extraction of dried *Lycium barbarum* also has strong antimicrobial and antifungal effects against various foodborne bacteria (Gram-, *Escherichia coli, Salmonella typhimurium, Campylobacter*), (Gram +, *Staphylococcus aureus, Listeria monocytogenes, Clostridium perfringens*), yeasts (*Yarrowia lipolytica, Metschnikowia fructicola, and Rhodotorula mucilaginosa*), and fungi (*Penicillium expansum, Aspergillus niger, Fusarium oxysporum, and Rhizoctonia solani*). Aqueous extracts with low maltodextrin and high phenolic content showed the highest activity. The polyphenol content, low maltodextrin concentration, and optimized ultrasonic extraction were linked to the antimicrobial effects. Aqueous extracts had higher inhibitory concentrations compared to ethanol or ethanol/hexane extracts. The quantity of maltodextrin used for encapsulation was critical, with a reduction from 14 % to 7 % enhancing antimicrobial activity. Aqueous extracts with higher total phenolic content also had stronger antimicrobial activity [69].

A different study revealed that the ethanolic extract of *Lycium barbarum* (LBE) displayed antimicrobial, antiadhesive, antibiofilm, and cytotoxic characteristics against common oral and periodontal pathogens. The antimicrobial impacts of LBE on five potential periodontal pathogens (*Porphyromonas gingivalis, Aggregatibacter actinomycetemcomitans, Fusobacterium nucleatum, Prevotella intermedia, Tanerella forsythia*) were examined and contrasted with chlorhexidine and doxycycline. LBE effectively hindered the growth of periodontal pathogens, although the inhibition zone was smaller compared to doxycycline and chlorhexidine. Notably, LBE exhibited the largest inhibition zone for *Tanerella forsythia* and *Aggregatibacter actinomycetemcomitans*. Furthermore, evaluations of cytotoxicity and cell viability of LBE on gingival fibroblast and modified keratinocyte lines demonstrated compatibility at a concentration of 1 mg/mL [70].

*Lycium barbarum* oligosaccharides, obtained through acid and enzymatic hydrolysis, possess antibacterial properties against *Staphylococcus aureus*. The increase in concentration of these oligosaccharides hindered the growth of *S. aureus*, demonstrating their antibacterial effect. This was confirmed through the growth curve and pH value of *Staphylococcus aureus*, measured using an ultraviolet spectrophotometer. However, it was noted that *Lycium barbarum* oligosaccharides were challenging to digest and absorb into gastrointestinal fluid [71].

The research on the antimicrobial properties of endophytic bacteria *Lycium barbarum* from Ningxia Province, China, utilizing 16S rRNA gene sequencing and agar diffusion to investigate the diversity and antimicrobial impacts of endophytic bacteria present in different parts of *Lycium barbarum* produced favorable outcomes. *Bacillus* spp. was recognized as the most common species throughout all tissues. The study revealed that 76.5 % of the samples exhibited antimicrobial activity against at least one of the five pathogenic bacteria examined, with *Bacillus* R2, R7, L3, and Brevundimonas R3 showing notable antimicrobial effects against *Colletotrichum nigrum* and *Exserohilum turcica*. However, the majority of the samples displayed a weak inhibitory effect on *Escherichia coli* and *Staphylococcus aureus* [72,73].

## 4 Anti-inflammatory activity

The study by Yang et al. illustrated the anti-inflammatory effects of *Lycium barbarum* fruits (LBF). The investigation demonstrated that LBF effectively lowered the levels of nitric oxide and proinflammatory cytokines in RAW264.7 macrophage cells exposed to lipopolysaccharides. The findings indicated a notable decrease in NO production at 400 μg/mL of LBF. Additionally, LBF displayed a dose-dependent suppression of NO production and a decrease in TNF-α, IL-6, and IL-1β concentrations post LPS stimulation. LBF exhibited promising anti-inflammatory properties by regulating the secretion of inflammatory mediators and factors during inflammation, by inhibiting TNF-α, IL-6, and IL-1β gene transcription [74].

The blend of *Chrysanthemum morifolium* flower heads (C) and *Lycium barbarum* fruits (*Lycium barbarum*, W) exhibited notable anti-inflammatory properties in RAW 264.7 macrophages when mixed in a 1:1 proportion. This combination decreased nitric oxide production and hindered the expression of inflammatory markers like iNOS, TNF-α, IL-1β, and IL-6 mRNA. In comparison to LPS treatment alone, CW decreased NO release by as much as 60 %, surpassing other compositions and matching the positive control dexamethasone. The suppression of iNOS expression by CW implies its involvement in reducing NO production. Furthermore, CW infusion efficiently deactivated MAPKs (ERK and JNK) and NF-κB, with a dose-dependent decline in inflammatory mediators. The presence of bioactive compounds in CW infusion, such as chlorogenic acid and *Lycium barbarum* polysaccharides, target various segments of the signal transduction pathway, contributing to its synergistic anti-inflammatory effects [75].

*Lycium barbarum* seeds and leaves possess anti-inflammatory properties that can effectively reduce the production of various inflammatory markers. Both extracts from *Lycium barbarum* seeds and leaves have demonstrated the ability to inhibit the expression of iNOS and COX-2. Moreover, the group treated with *Lycium barbarum* leaf extract showed significantly lower TNF-α production compared to the control group, while IL-6 production notably decreased in the chlorophyll-removed *Lycium barbarum* leaf extract group. Although not statistically significant, serum IL-1β concentrations tended to decrease with *Lycium barbarum* leaf extract compared to LPS alone. The comet assay results revealed that *Lycium barbarum* leaf extracts effectively prevent DNA damage in lymphocytes, as evidenced by significantly lower values for tail DNA, tail length, and tail moment compared to the LPS only group (p < 0.05) [76]. Furthermore, a study by Bae et al. also illustrated the anti-inflammatory properties of *Lycium barbarum* seed extract (LFE), leaf extract (LLE), and chlorophyll-removal leaf extract (LLE with CR). The results showed that LFE, LLE, and LLE with CR effectively reduced the production of pro-inflammatory mediators (NO, TNF-α, IL-6, and IL-1β) as well as iNOS and COX-2 expression in LPS-stimulated RAW 264.7 cells in a dose-dependent fashion. Moreover, the administration of LLE and LLE with CR led to decreased levels of proinflammatory cytokines in the bloodstream and minimized DNA damage in BALB/c mice. Notably, LLE with CR displayed the most potent anti-inflammatory effects [77]. At the same time, the combination of 50 % ethanol fractions of *Lycium barbarum* with chlorophyll-removal leaves in different ratios had inhibitory effects on the production of NO, TNF-α, and IL-6 in Raw 264.7. Notably, samples with ratios of 3:1 and 5:1 showed significant inhibition of these inflammatory markers in lipopolysaccharide-stimulated Raw 264.7 cells. Interestingly, mixtures with a higher proportion of chlorophyll-removal *Lycium barbarum* leaves demonstrated a more potent anti-inflammatory effect [78].

The fruit of *Lycium barbarum* has demonstrated anti-inflammatory properties in Wistar rats exposed to lipopolysaccharide (LPS)-induced systemic inflammation. The analysis showed that the group fed a standard diet + water + LPS had the highest inflammatory response, while the group receiving the palatable diet along with the fruit extract showed a reduction in inflammation compared to the group receiving the palatable diet alone. Additionally, there was a decrease in the activity of glutamine-oxaloacetic transaminase in the *Lycium barbarum*-fed groups compared to the water-fed groups, and an increase in creatinine levels in the water-fed group compared to the L. barbarum-fed groups. The expression of inflammation marker genes in the liver also showed significant differences between the groups [79]. Additionally, a study conducted in live animals to explore the possible metabolic advantages of *Lycium barbarum* supplementation in counteracting obesity and its related conditions caused by a high fat diet, revealed that both *Lycium barbarum* and fermented *Lycium barbarum* with *L. plantarum* CB3 supplementation reduced the activity of genes associated with inflammation at both local and systemic levels in rats. Moreover, supplementation with both types of *Lycium barbarum* also shielded against liver damage and abnormal lipid levels caused by a high fat diet, mainly by enhancing liver function and lipid metabolism due to their anti-inflammatory properties [80].

*Lycium barbarum* polysaccharides possess anti-inflammatory and analgesic properties, as demonstrated in various tests such as the hot plate test, formaldehyde pain experiment, and primary cultured dorsal root ganglion neuronal cells in mice. The polysaccharides were shown to significantly reduce phase II pain caused by formalin injection, as well as decrease hind leg weight difference and levels of IL-6 in the blood and spinal cord tissue. It is interesting to note that *Lycium barbarum* polysaccharides did not affect hot plate latency in mice. Furthermore, they were found to decrease TRPV1 function on primary cultured DRG cells. The anti-inflammatory and analgesic effects of *Lycium barbarum* polysaccharides are thought to be associated with a decrease in IL-6 expression and an increase in TRPV1 activity [81]. Simultaneously, a hemostatic hydrogel containing functionalized *Lycium barbarum* polysaccharide (LBP) loaded ultrathin MMT nanosheets (L-MMT NS) demonstrated remarkable hemostatic properties in a mouse liver hemorrhage model, while also minimizing tissue damage from inflammation and accelerating the wound healing process [82]. On the other hand, *Lycium barbarum* polysaccharides (LBP, 51.25 % polysaccharides) and capsaicin (CAP, 85 % capsaicin) from cayenne pepper extract exhibited protective effects against sodium dextran sulfate (DSS)-induced colitis in rats. LBP, CAP, and the combination of both (MIX) significantly improved disease activity index (DAI) scores on days 5 and 6, reversed colon length reduction, and decreased mass-to-length ratio caused by DSS. IFN-γ, IL-17A, and IL-22 levels in the colon were notably reduced by LBP and CAP, with only IL-22 levels significantly reduced in the MIX group. These results indicate that LBP and/or CAP can mitigate IBD symptoms and enhance colon health by reducing inflammation [83]. Simultaneously, *Lycium barbarum* polysaccharides (LBP) exhibited promising outcomes in diminishing inflammation and oxidative stress in mice suffering from acute pancreatitis (AP). The research revealed that LBP lessened the pancreatic coefficient, enhanced the pathology score of pancreatic tissue, reduced levels of serum IL-6, IL-β, and TNF-α, as well as decreased MDA and MPO levels in the pancreas. Moreover, SOD content rose while tissue diminished, and there was a notable decline in the expression of mRNA p65 and protein. The results propose that the mechanism is linked to the suppression of inflammatory factors, oxidative stress molecules, and the NF-κB signaling pathway [84].

The research on the impact of *Lycium barbarum* (LF) on endoplasmic reticulum (ER) stress revealed that the LF extract increased the size of the thyroid gland, regardless of the presence of inflammation. When Caco-2 cells were treated with LF, the levels of the pro-inflammatory marker IL-8 did not show a significant change, but the expression of the ER stress marker XBP1 notably increased. In wild-type (wt) MEF cells, exposure to 12.5–50 μg/mL of the extract led to a dose-dependent increase in spliced forms of IL-6, CHOP, and XBP1. However, the absence of XBP1 or IRE1α in MEF cells abolished this effect. LF treatment improves barrier function, reduces inflammation, and alleviates ER stress in an IRE1α-XBP1-dependent manner [85].

## 5 Prebiotic activity

*Lycium barbarum* polysaccharide (LBP) has shown great potential as a prebiotic, with the ability to positively impact gut microbiota by increasing levels of beneficial bacteria and influencing the immune response. Studies on *Lycium barbarum* polysaccharide (LBP) have demonstrated its capacity to support the growth of specific probiotic bacteria, with significant increases observed in *Lactobacillus acidophilus* and *Bifidobacterium longum* in the fecal microbiota of mice. The introduction of LBP led to changes in the composition of different phyla and genera, promoting the growth of potential probiotic genera like *Akkermansia*, *Lactobacillus*, and *Prevotellaceae*. Furthermore, the administration of LBP resulted in elevated levels of certain immune markers in the serum and colonic contents of mice. Notably, there were significant differences in the thymus index and spleen index of mice treated with LBP compared to the control group [86]. Moreover, the combination of *Lycium barbarum* polysaccharide (LBP) and *Laminaria japonica* polysaccharide (LJP) has been recognized as an effective prebiotic for regulating intestinal probiotics and their metabolites. The different combinations of LBPs and LJPs showed superior prebiotic effects compared to individual LJPs, with the 4:1 ratio of LBP and LJP extracted at 50 °C displaying the most potent effect. The optimal compound polysaccharide delivered exceptional results and complementary functions by promoting the proliferation of *Bifidobacterium*, *Lactobacillus*, and *Bacteroides* through LBP, as well as the production of SCFAs and health-related non-SCFA metabolites [87].

Fermented counterpart (FGB) of *Lycium barbarum* (GB) demonstrated prebiotic properties that can enhance gut barrier function and decrease inflammation related to metabolic issues. A study conducted Jeong et al., examining the effects of GB and FGB supplementation on gut health, inflammation, and changes in gut microbiota in high-fat (HF) rats found significant changes in gut microbiota composition. Specifically, GB supplementation led to an increase in *Faecalibacterium prausnitzii* species, while FGB supplementation resulted in higher levels of *Akkermansia muciniphila* species. These changes were associated with improvements in obesity-related characteristics and metabolic parameters. Additionally, the improved gut integrity observed with GB and FGB supplementation acted as a defense against inflammation triggered by the HF diet, including the reduction of lipopolysaccharide-induced inflammation by inhibiting its downstream pathway [88]. However, the incorporation of functional (prebiotic) white chocolate enriched with *Lycium barbarum* as an antioxidant and sucrose alternative resulted in a decrease in the perception of most aroma and flavor characteristics, while enhancing bitter taste, bitter aftertaste, astringency, and various texture attributes. Nevertheless, consumers still approved of the chocolate across all sensory aspects, with satisfaction ratings exceeding 6 on a 9-point scale. The findings from partial least squares PLS regression analysis showed that creamy color and cocoa butter flavor descriptors positively influenced the acceptance of functional white chocolate [89].

Analysis using scanning electron microscopy (SEM) and atomic force microscopy (AFM) revealed that the bioactive components of LBP can blend with gluten protein, enhancing the noodle structure and strengthening gluten cross-linking. The findings indicated that the addition of LBP improved the gluten protein and the starch-gluten composite system overall, although it led to a decrease in starch digestibility. Incorporating LBP, typically at levels below 5 %, can enhance the performance and structure of gluten proteins. Additionally, LBP had a notable effect on surface hydrophobicity (ranging from 0 % to 5 %), with the concentration of free thiol groups increasing from 1.93 μmol/g to 4.19 μmol/g. Furthermore, the addition of LBP positively influenced the gluten structure, resulting in improved texture, elasticity, cooking properties, and flavor of the noodles. Hardness and recovery significantly increased by approximately 25.97 % and 11 % (p < 0.05), while cooking yield and cooking losses also showed significant changes (p < 0.05) [90].

A study was conducted to prepare chocolate with lower saturated fatty acid content and higher nutrient content using beeswax complex oleogels with glycerol monostearate (GBO), rice bran wax complex oleogels with glycerol monostearate (GRO) and lac wax complex oleogels with glycerol monostearate (GLO)), and also using Lycium barbarum seed oil as a base oil with wax showed that among the three oleogels, GRO showed the highest hardness and oil binding capacity (OBC) of 1457 g and 99.82 %, respectively. At the same time, GRO demonstrated the best properties gelation at 1–100 Hz and 0.001–10 % sweep strain. Oleogellated chocolates were characterized for rheology, crystal polymorphism, and sensory properties. Compared to the control group without oleogels, GRO showed better replacement, with GRO chocolate having more stable rheological properties, better taste and overall acceptability [91].

## 6 Neuroprotective activity

The aqueous extract of *Lycium barbarum* berries has been shown to protect neurons by reducing oxidative stress and neuroinflammation. A study on BALB/c mice found that daily oral administration of this extract for 4 weeks after acute irradiation improved weight loss, radiation-induced depressive behavior, spatial memory impairment, and hippocampal neuronal loss, indicating the potential benefits of *Lycium barbarum* berry extract in addressing neurobehavioral changes. The research demonstrated positive effects on hippocampal neurons, leading to improvements in cognitive decline and depressive symptoms caused by radiation exposure [92].

*Lycium barbarum* polysaccharides (LBP) had a beneficial impact on depressive-like behavior in ovariectomized rats. The combination of low-dose LBP and imipramine resulted in a reduction in depressive-like behavior through an increase in serum superoxide dismutase activity and a decrease in serum malondialdehyde levels. Furthermore, it was discovered that low-dose LPB, high-dose LBP, and imipramine led to an increase in the quantity of 5-HT2A receptors and BDNF-positive cells, while also decreasing the number of TUNEL-positive cells in the hippocampus [93]. Additionally, an in vitro study revealed that a water-soluble *Lycium barbarum* polysaccharide, composed of arabinose and galactose, caused a concentration-dependent decrease in Aβ42/Aβ40 levels in N2a/APP695 cells [94]. Moreover, studies have shown that utilizing *Lycium barbarum* polysaccharides can boost neuroplasticity in the prefrontal cortex and hippocampus, key regions in the aging brain. An examination on the impact of *Lycium barbarum* on recognition memory and dendrite morphology in elderly rats revealed that those given *Lycium barbarum* displayed notable enhancements in dendritic architecture in the prefrontal cortex and hippocampus. Additionally, these rats displayed increased levels of synaptophysin, decreased reactive astrogliosis, and reduced levels of caspase-3, 3-NT, and Nrf2 in these brain areas [95]. On the other hand, *Lycium barbarum* polysaccharides (LBP) exhibit a neuroprotective effect on light-induced neuronal damage, improving cognitive function and reducing depressive symptoms in mice exposed to LAN. LBP also mitigated light-triggered apoptosis and mitochondrial injury in HT-22 cells, while preserving mitochondrial membrane permeability levels. Moreover, LBP administration reversed the suppression of Nrf2/HO-1 signaling pathways in light-exposed mice and cells [96]. Additionally, a study on the neuroprotective properties of *Lycium barbarum* polysaccharides (LBP) in glaucomatous retinal neuropathy using a rat model of high intraocular pressure (HIOP)-induced retinal ischemia found that LBP showed great antioxidant effects in the treated groups. Specifically, LBP at a dose of 250 mg/kg showed significant retinal neuroprotection by preserving retinal structure and function while boosting antioxidant capacity [97].

The study of the neuroprotective effects of methanolic *Lycium barbarum* fruit extract (LBFE) using a Drosophila PD model (PINK1B9) and the human neuroblastoma cell line SH-SY5Y found that *Lycium barbarum* has pharmacological properties, such as anti-aging and antioxidant effects related to Parkinson's disease, and plays a vital role in maintaining neuronal cell homeostasis. Giving LBFE to PINK1B9 flies at 6, 12, and 18 days of age led to increased ATP and dopamine levels at all ages, prolonged lifespan, enhanced locomotor behavior, and reversed olfactory deficits in PINK1B9 flies. Additionally, histopathological studies showed a significant restoration of muscle atrophy in the thorax of mutant flies. LBFE also demonstrated the ability to protect SH-SY5Y cells from MPP + -induced neurotoxicity [98].

## 7 Antidiabetic activity

*Lycium barbarum* polysaccharides (LBP) can potentially improve blood sugar levels and β-cell function in diabetic mice by impacting the gut microbiota and intestinal barrier. A study on a mouse model of high-fat and streptozotocin-induced diabetes showed that six weeks of LBP oral administration led to a 13.51 % reduction in fasting blood glucose levels. Additionally, there was a 3.3-fold increase in a specific taxon of the genus *Allobaculum* in the intestinal bacterial community. Further research involving fecal microbiota transplants and antibiotic treatment confirmed the role of LBP-mediated gut microbiota in glycemic control for diabetes treatment. Moreover, LBP intervention was found to boost zonula occludens 1 expression, thereby safeguarding intestinal barrier function both in vivo (assay of intestinal permeability in diabetic mice) and in vitro (using a Caco-2/RAW264.7 intestinal-like cell co-culture inflammation model) [99]. The utilization of LBP resulted in notable enhancements in a variety of physical parameters and metabolic processes in mice with HFD/STZ-induced T2DM. These improvements included increased body weight, GLP-1 level, water intake, liver index, fasting blood glucose, HOMA-IR, HOMA-IS, HbA1c, OGTT levels, as well as decreased TC and TG levels in both serum and liver. Furthermore, LBP suppressed the expression of specific mRNAs, reduced pro-inflammatory cytokines, and restored the liver tissue structure. LBPs have demonstrated efficacy in ameliorating HFD/STZ-induced T2DM in mice by targeting three main areas: enhancing glucose and lipid metabolism, inhibiting pro-inflammatory cytokines, and modulating gut microbiota [100].

A research study on the inhibition of high glucose-induced angiogenesis by *Lycium barbarum* polysaccharides (LBP) in RF/6A cells found that LBP can reduce diabetic retinal angiogenesis by restoring miR-15a-5p expression in RF/6A cells. The study involved subjecting RF/6A cells to high glucose-induced angiogenesis for 48 h, followed by an additional 48 h in high glucose (25 mM). The results showed a significant decrease in miR-15a-5p and miR-15a-3p expression after exposure to high glucose levels of 25 or 50 mmol/L, with miR-15a-5p levels being higher than miR-15a-3p. Treatment with miR-15a-5p LBP at 600 mg/L resulted in increased cell apoptosis and total length of vascular branches. Additionally, the protein expression of VEGFA, VEGFR2, and ANG2 decreased, while ANG1 expression increased. Furthermore, ASM mRNA and protein expression were also downregulated [101].

A study conducted by Lei et al. found that *Lycium barbarum* polysaccharides (LBP) protected testicular function in diabetic rats induced by streptozotocin (STZ). The diabetic rats had higher blood glucose levels and insulin resistance compared to the control group, leading to decreased testicular function. Treatment with metformin and LBP improved blood glucose levels, insulin resistance, and testicular function in diabetic rats. LBP displayed protective effects by stimulating cell proliferation, inhibited cell apoptosis, and regulated SIRT1/HIF-1α expression in the testes of diabetic rats [102].

*Lycium barbarum* polysaccharides (LBP) exhibits hypoglycemic and renal protective effects in mice with high-fat diet/streptozotocin-induced diabetic nephropathy, suppressing inflammation through the inhibition of NF-κB activation. It also reduces glucose levels, improves insulin resistance, and enhances renal function by decreasing sCr, BUN, and microalbumin levels, while mitigating glomerular and tubular damage. LBP also diminishes renal inflammation by lowering TNFα, IL1β, IL6, and SAA3 mRNA levels in the renal cortex, as well as reducing elevated circulating levels and renal SAA3 protein deposits. Furthermore, LBP inhibits the activated signaling of nuclear translocation of NF-κB p65 and degradation of IκBα [103]. Zhang & Tang's research also discovered that *Lycium barbarum* polysaccharides (LBP) has a significant protective impact on diabetic kidney damage. In a streptozotocin-induced rat model of diabetes, there was an increase in blood glucose levels, Scr, BUN, CRP, IL-6, and TNF-α in the blood serum. Furthermore, MDA content increased in kidney tissue while GSH content increased. Conversely, the activity of GSH-Px, CAT, and SOD decreased, and the expression levels of NF-κB p65 subunit, MCP-1, and ICAM-1 increased. LBP treatment resulted in reduced blood glucose levels, serum levels of Scr, BUN, CRP, IL-6, TNF-α, and MDA content in kidney tissue. It also increased GSH, GSH-Px, CAT content, SOD, and suppressed the expression levels of NF-kB p65 subunit, MCP-1, and ICAM-1 compared to the diabetic group. The mechanism of action involves improving blood glucose levels, reducing renal oxidative stress, and decreasing inflammatory response in diabetic rats [104].

Liposomal *Lycium barbarum* polysaccharide nanoparticles enhance the recovery of chronic wounds in diabetic feet by influencing the CXCL12/CXCR4 signaling pathway. In mice treated with *Lycium barbarum* polysaccharide liposomal nanoparticles, there was a decrease in blood sugar and the inflammatory factor IL-6, leading to a significant improvement in wound healing (P < 0.05). The *Lycium barbarum* lipid nanoparticle-polysaccharide nanocomposite (LNP-LBP) also reduced the levels of CXCL12 and CXCR4 in mouse wound tissues (P < 0.05). Additionally, the combination of LNP-LBP and CXCL12/CXCR4 signaling pathway inhibitors further accelerated the wound healing process and increased IL-6 levels. LNP-LBP also decreased the blood sugar level in diabetic rat feet, diminishing the inflammatory response and swelling of wound podocytes, and promoting cellular autophagy to enhance metabolism, ultimately assisting in the healing of persistent diabetic foot wounds [105].

*Lycium barbarum* polysaccharides (LBP) have a protective effect on neuronal axonal injury induced by high glucose. In both in vivo and in vitro experiments, it was observed that hippocampal tissue in a rat model of STZ-induced type II diabetic encephalopathy displayed damaged neuronal axons and hyperphosphorylation of the Tau protein, resulting in axonal transport damage and cognitive dysfunction. LBP significantly reduced peripheral blood glucose and serum insulin levels in the rat model of type II diabetic encephalopathy, leading to decreased peripheral insulin resistance. Additionally, LBP improved learning and memory deficits, as well as neuronal axonal pathology in the rat model. Treatment with LBP effectively inhibited the PI3K/Akt signaling pathway and reduced tau protein phosphorylation levels by activating the PI3K/Akt/GSK3β signaling pathway, ultimately offering protection against neuronal axonal damage and enhancing cognitive function in rats with type II diabetic encephalopathy [106].

*Lycium barbarum* glycopeptide demonstrated a notable impact on improving anti-inflammatory, antioxidant stress, and anti-apoptosis in diabetic retinopathy mice through the p-Akt and p-PI3K signaling pathways. The administration of *Lycium barbarum* glycopeptide led to a substantial rise in retinal thickness in diabetic mice, assisting in alleviating the histopathological changes associated with diabetic retinopathy. Furthermore, the glycopeptide effectively reduced the levels of proinflammatory cytokines (TNF-α, iNOS, IL-6, IL-1β, and Nrf2) induced by diabetic retinopathy in the retina, as confirmed by qPCR analysis. GFAP immunostaining revealed increased intensity in the retinas of diabetic mice, a response that was counteracted by *Lycium barbarum* glycopeptide treatment. Western blot analysis showed that LBGP treatment decreased caspase-3 levels and increased Bcl2 expression. Noteworthy, the expression of phospho-Akt (p-Akt) and phospho-PI3K (p-PI3K) was significantly heightened in the retinas of diabetic mice, a effect that was reversed by LBGP administration [107].

## 8 Gastroprotective effects

Pre-treatment with *Lycium barbarum* polysaccharides (LBP) and/or C-PC resulted in a reduction in the severity of ethanol-induced gastric ulcers in rats by inhibiting oxidation and inflammation, while also improving gastroprotection. After 5 weeks of treatment, histopathological analysis revealed that either LBP or C-PC decreased the extent of damage to the gastric mucosa. LBP notably lowered serum malondialdehyde (MDA), gastric interleukin-6 (IL-6), intercellular adhesion molecule-1 (ICAM-1), and myeloperoxidase (MPO) activity. Meanwhile, C-PC reduced serum MDA levels and gastric tumor necrosis factor-α (TNF-α) levels, IL-1β, IL-6, ICAM-1, and MPO activity. The combined use of LBP and C-PC led to decreased serum MDA levels and gastric TNF-α, IL-1β, IL-6, and ICAM-1 levels. Additionally, LBP and/or C-PC raised the levels of gastric heat shock protein 70 and non-protein sulfhydryl compounds [108]. In addition, the combination of LBP and C-phycocyanin (CPC) from Spirulina platensis displayed protective effects against aspirin-induced gastric ulcers in rats. The blend improved gastroprotective factors, inhibited lipid peroxidation, and increased the presence of gastric bifidobacteria. The rats that received the LBP and CPC mixture showed a significant rise in gastric cyclooxygenase-1, prostaglandin E2, and total nitrite and nitrate levels by 139 %, 86 %, and 66 %, respectively, compared to the aspirin group (p < 0.05). Moreover, this group decreased lipid peroxides and malondialdehyde levels by 78 % (p < 0.05). Administration of BNB and/or COD resulted in a 2.5–4.0 times elevation in the relative abundance of gastric bifidobacteria compared to the aspirin group (p < 0.05) [109]. In a study with similar parameters, the impact of CPC and/or LBP on aspirin-induced gastric injury was investigated in rat gastric mucosal RGM-1 cells. CPC and/or LBP increased IL-10 and mitigated pro-inflammatory processes and markers, Bax protein, NF-κB, and ERK and JNK activation. CPC and/or LBP exhibit anti-inflammatory effects by inhibiting the activation of the ERK signaling pathway, and LBP reduces apoptosis by suppressing the activation of the JNK signaling pathway in gastric RGM-1 cells with aspirin-induced epithelial damage [110].

The study by Wang et al. demonstrated that *Lycium barbarum* polysaccharides (LBP) have the potential to hinder the growth of gastric cancer cells. The results indicated that LBP exerts its anti-cancer effects by influencing the miR-202-5p/PIK3CA axis in gastric cancer. Treatment with LBP led to a decrease in the survival of GC cells, with the impact varying depending on the dosage and duration of treatment. Additionally, LBP treatment resulted in a significant reduction in cell growth and movement, while increasing the rate of cell death in GC cells. The increase in caspase-3/7 and miR-202-5p levels in GC cells due to LBP directly affected PIK3CA, as confirmed by luciferase assay and the ability of anti-miR-202-5p to reverse LBP's inhibitory effects on PIK3CA. Furthermore, LBP was found to decrease the levels of downstream targets of PIK3CA, such as AKT and mTOR, by enhancing miR-202-5p. The anti-cancer properties of LBP in GC cells are associated with the increased expression of miR-202, which disrupts the PIK3CA/AKT/mTOR pathway [111].

*Lycium barbarum* polysaccharides (LBP) effectively reduces UC by modulating inflammatory cytokines, promoting probiotic growth, inhibiting harmful pathogens, and increasing SCFA production. It improves colitis by increasing body weight, colon length, reducing DAI, and enhancing histopathological parameters. LBP downregulates pro-inflammatory cytokines (IL-1β, IL-6, iNOS, and TNF-α) and upregulates anti-inflammatory cytokines (IL-10), promotes tight junction proteins (occludin and ZO-1), and protects the intestinal mucosal barrier. It increases specific probiotics (*Ruminococcaceae*, *Lactobacillus*, *Butyricicoccus*, and *Akkermansia*) and reduces opportunistic pathogens (*Mucispirillum* and *Sutterella*) associated with UC, while enhancing SCFA production, including acetic acid, propionic acid, butyric acid, and isobutyric acid [112].

To assess the effect of the digestive system on the phenolics and antioxidant capacity of *Lycium barbarum* fruit, its extract was digested and analyzed in vitro. The results revealed that the phenolics/flavonoids contents and antioxidant capacity in the pH-treated group and digested group generally decreased by 4.39%–21.22 % in the oral and intestinal digestions but increased by 1.26%–16.14 % in the gastric digestion. The phenolic content and antioxidant capacity were highly correlated in digested groups (r > 0.899, p < 0.01). Five main phenolics including rutin, p-coumaric acid and its hexoside derivatives were identified and quantified, and these phenolics displayed higher stability during the digestion, implying that they may be the potential functional phenolics of *Lycium barbarum* fruit [113].

## 9 Conclusion

The most recent review summarizes the newest research discoveries on the extraction, refinement, and various forms of LBPs, as well as their pharmacological properties. *Lycium barbarum* has a lengthy history as a medicinal and functional food. The *Lycium barbarum* has gained attention from both domestic and international markets in recent years due to its high-quality health benefits and significant potential for development. It contains a variety of active ingredients such as polysaccharides, polyphenols, flavonoids, and betaine, which have antioxidant, immune regulation, hypoglycemic, and vision improvement properties. However, the development of *Lycium barbarum* still requires improvement in several areas: new types of nanoliposomes and microcapsules of *Lycium barbarum* should be developed in the future to protect sensitive compounds and enhance therapeutic effects, and further research on fermentation technology could be integrated into artificial intelligence, such as machine learning and artificial neural network. This would allow for timely adjustments to fermentation parameters based on the detection of substances such as sugars and proteins during the fermentation process, ensuring the quality and safety of the products. Despite the fact that the valorization of goji by-products helps reduce food waste during processing, the high energy and solvent usage in extraction processes remain challenges. The implementation of green extraction techniques at industrial levels in the future is expected to minimize waste and maximize the extraction of bioactive compounds from *Lycium barbarum* and by-products.

## CRediT authorship contribution statement

**Baiken Baimakhanova:** Conceptualization. **Amankeldi Sadanov:** Conceptualization. **Andrey Bogoyavlenskiy:** Conceptualization. **Vladimir Berezin:** Formal analysis. **Lyudmila Trenozhnikova:** Formal analysis. **Gul Baimakhanova:** Formal analysis. **Aibat Ibraimov:** Formal analysis. **Elmira Serikbayeva:** Formal analysis. **Zhalgaskali Arystanov:** Writing – original draft. **Tanagul Arystanova:** Writing – original draft. **Rakhym Nazakat:** Formal analysis. **Ainura Khammetova:** Writing – original draft. **Gulnaz Seitimova:** Writing – review & editing. **Aknur Turgumbayeva:** Writing – review & editing.

## Institutional review board statement

Not applicable.

## Informed consent statement

Not applicable.

## Data availability statement

Not applicable.

## Funding

This work was funded by Ministry of Science and Higher Education, Republic of Kazakhstan. Grant number [BR21882248].

## Declaration of competing interest

The authors declare the following financial interests/personal relationships which may be considered as potential competing interests:

This work was funded by Ministry of Science and Higher Education, Republic of Kazakhstan, Grant number [BR21882248]. LLP “Research and Production center for Microbiology and Virology”, 105 Bogenbay Batyr Str, Almaty, 005010, Kazakhstan.
